# Oral health problems among palliative and terminally ill patients: an integrated systematic review

**DOI:** 10.1186/s12903-020-01075-w

**Published:** 2020-03-18

**Authors:** Munikumar Ramasamy Venkatasalu, Zaidah Rizidah Murang, Divya Thirumalai Rajam Ramasamy, Jagjit Singh Dhaliwal

**Affiliations:** Pengiran Anak Puteri Rashidah Sa’adatul Bolkiah Institute of Health Sciences, Universiti Brunei Darussalam, Bandar Seri Begawan, Brunei Darussalam

**Keywords:** Oral conditions, Oral diseases, Palliative, Terminally-ill, Cancer, Integrated review

## Abstract

**Background:**

High incidence of treatable oral conditions has been reported among palliative patients. However, a large proportion of palliative patients lose their ability to communicate their sufferings. Therefore, it may lead to under-reporting of oral conditions among these patients. This review systematically synthesized the published evidence on the presence of oral conditions among palliative patients, the impact, management, and challenges in treating these conditions.

**Methods:**

An integrative review was undertaken with defined search strategy from five databases and manual search through key journals and reference list. Studies which focused on oral conditions of palliative patients and published between years 2000 to 2017 were included.

**Results:**

Xerostomia, oral candidiasis and dysphagia were the three most common oral conditions among palliative patients, followed by mucositis, orofacial pain, taste change and ulceration. We also found social and functional impact of having certain oral conditions among these patients. In terms of management, complementary therapies such as acupuncture has been used but not well explored. The lack of knowledge among healthcare providers also posed as a challenge in treating oral conditions among palliative patients.

**Conclusions:**

This review is first in its kind to systematically synthesize the published evidence regarding the impact, management and challenges in managing oral conditions among palliative patients. Although there is still lack of study investigating palliative oral care among specific group of patients such as patients with dementia, geriatric or pediatric advanced cancer patients, this review has however provided baseline knowledge that may guide health care professionals in palliative settings.

## Introduction

High incidence of oral conditions were often reported among palliative patients either direct or indirect primary cause such as salivary gland dysfunction in non-Hodgkin’s lymphoma or fatigue which may affect patient’s ability to undertake oral care hygiene [1, 2]. Medical management of palliative conditions such as chemotherapy were often reported which can produce oral complications among these patients [2]. For example, the National Cancer Institute at the National Institutes of Health, United States of America reported that 80% of patients receiving myeloablative chemotherapy will develop oral complications, and palliative drugs such as bisphosphonates and analgesics were associated with oral mucositis and taste disturbance [3].

Early diagnosis and treatment of oral conditions among palliative patients could minimize their pain and suffering [2]. However, evidence shows that 40% of palliative patients lose their ability to communicate their oral health needs. Therefore, they may suffer treatable oral conditions for a prolonged period of time [4], or they may not complain of discomfort in their oral cavity which they believe to be an inevitable effect of their treatment [5]. This may contribute to under-reporting as well as underestimation of oral conditions among palliative patients, which may result in failure among health professionals to completely appreciate the problem. A literature review of oral care for cancer patients in 2001 reported that oral care is given by junior staffs with less experience and the practice needs to be transferred to oncology nurses [6]. Furthermore, a survey of international supportive health care providers (*n* = 212) (with 35% response rate) recommended to develop evidence-based practice protocol for oral care management [7].

This systematic review aimed to synthesize the published evidence on oral conditions among palliative patients, impact, management and challenges in managing oral conditions among palliative patients.

## Materials and methods

### Data sources

Search strategy was devised by the research team with chosen five databases in specific period in English language with comprehensive search terms to not omit any relevant potential primary studies. The detailed data sources are explained in Table 1.

**Table 1:** Details of data sources

Research team	A palliative nurse (MRV) A dentist (JSD) A medical doctor (DR) \ A healthcare researcher (ZR)
Data bases	Sciencedirect, PubMed, Google scholar, Ovid and EBSCOhost
Other resources	Reference list and manual search in key journals
Search time	January 2000 to December 2017
Language	Primary studies in English language
Search terms	“oral condition” OR “oral disease” OR “dental disease” OR “mouth disease” OR “mouth condition” OR mucositis OR stomatitis OR candidiasis OR cheilitis OR xerostomia OR “periodontal disease” OR halitosis OR thrush OR “angular cheilitis” OR “denture stomatitis” OR gingivitis OR periodontitis OR “mouth ulcer” OR “aphthous ulcer” AND palliative OR terminally-ill OR “terminally ill” OR “advanced disease” OR “advanced illness” OR dying OR end-of-life OR hospice OR cancer AND treatment OR intervention OR therapy OR management OR “oral care” OR “mouth care” OR “dental management” AND “end-of-life care”

### Study selection

Inclusion criteria specified that studies must be: (1) in full-text, (2) published between years 2000 to 2017, and (3) primary articles focusing on palliative patients and their oral conditions.

**Fig. 1:**
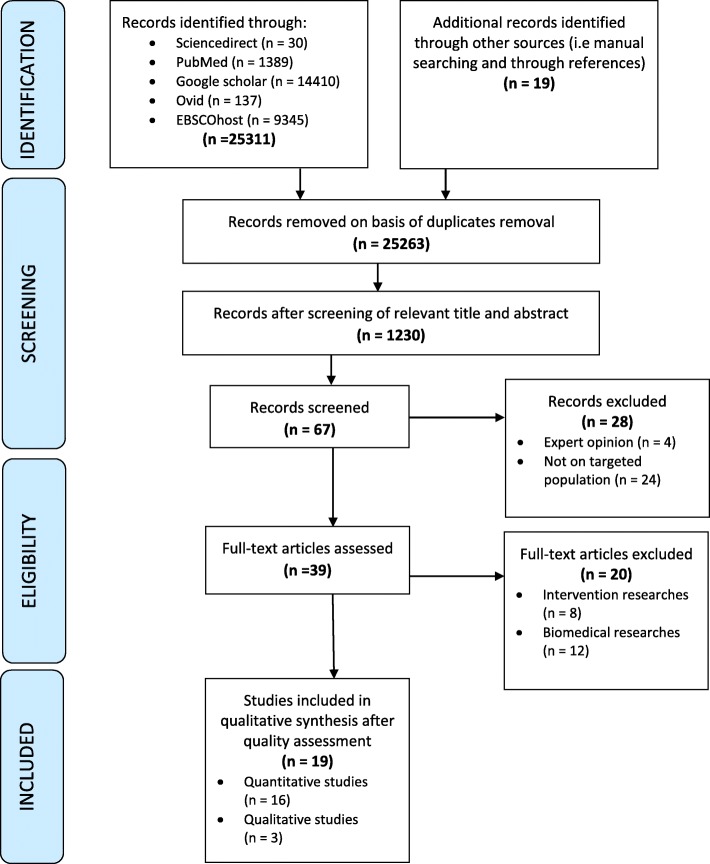
Process of studies selection

Figure 1 illustrates Preferred Reporting Items for Systematic Reviews and Meta-Analyses (PRISMA) flow chart of study selection process [8]. The initial combined search identified 25,311 articles from 5 databases and from other sources (manual searching and through references). Removal of duplicates resulted in 13,263 studies. Screening of relevant abstracts resulted in 1230 studies. Further screening for inclusion criteria resulted in 67 studies which were read to ensure applicability to our study. This resulted in 28 articles being excluded. All reviewers screened and discussed preliminary findings to reach a consensus on studies to be included that resulted in total of 19 articles for further analysis.

### Data extraction

In the data extraction process [9], study details were extracted into a table (Table 1). This was done by two reviewers (Z.R., and D.R.). All reviewers discussed each article to reach consensus regarding the study details. For each included study, the following information was extracted: author(s), year published, title, purpose, setting, participants, study design, and oral conditions present. The impact, management and challenges of oral problems among palliative patients were also extracted and summarized according to our research questions.

### Assessment of study quality

As our review included both qualitative and quantitative studies, we did not use any scoring for assessing the quality of studies included. Rather, the quality of the identified studies was assessed using Joanna Briggs Institute (JBI) critical appraisal tool [10]. As a result, only studies that were thoroughly appraised (have clearly defined inclusion criteria, study subjects and setting described in detail, exposure measured in a valid and reliable way, standard criteria used for measurement of condition, identification of confounding factors, outcomes measured in a valid and reliable way, and appropriate statistical analysis used) and agreed by all involved reviewers were included in this systematic review to write the findings.

### Data analysis

Extracted data from all included studies were analyzed using the Whittemore and Knafl [9] principles of integrative review with four stages: data reduction, data display, data comparison, and conclusion and verification. At data reduction stage, all 19 primary sources included in the integrative review were divided into subgroups; initially based on types of study (qualitative and quantitative), sample (cancer patients, non-malignant palliative conditions and oral conditions among palliative patients) and then by a predetermined conceptual classification aligning with the aims of this review and then analyzed by topic. Each primary source was reduced to a single page (available on request from authors). This helped us to systematically compare primary sources on specific issues, variables and sample characteristics. It also allowed us to organize the data into a manageable framework. At stage 2 (data display), the single page data from the 19 included studies was extracted and displayed in the form of a table (see Table 1). This helped us to visualize the patterns and relationships between and within primary data sources. At stage 3 (data comparison), we used constant comparison as a method of an iterative process of examining data to identify themes that had similar patterns and relations. Finally at stage 4 (conclusion and verification), patterns using primary data were verified and any similarities, differences and any spurious findings were identified, in order to ensure that valuable information was not lost. Five consecutive meetings were held in order to identify and reach a consensus on the final themes.

## Results

### Characteristics of study

Overall, 19 articles were included in this review. The majority of the participants were cancer patients (*n* = 14). Studies reporting on oral conditions among palliative patients are summarized in Table 1. Of the included studies, 16 were quantitative studies [5, 11–24], and 3 were qualitative studies [25–27](Table 2).

**Table 2:** Summary of included studies

Author(s)/Year	Title	Purpose	Setting	Participants	Study design	Oral conditions present
Nakajima /2017	Characteristics of Oral Problems and Effects of Oral Care in Terminally Ill Patients With Cancer	To investigate oral problems in terminal stage of cancer and improvement of dry mouth by oral care.	Japan	Terminally-ill cancer patients	Quantitative descriptive	1. Dry mouth 2. Stomatitis 3. Candidiasis
Fischer et al./2014	Oral health conditions affect functional and social activities of terminally ill cancer patients	To characterize oral conditions in terminally ill cancer patients to determine the presence, severity, and the functional and social impact of these oral conditions.	United States of America	Terminally-ill cancer patients	Quantitative descriptive	Using standardized oral examination: 1. Salivary hypofunction 2. Mucosal erythema 3. Ulceration 4. Fungal infection Using Oral Problem Scale (OPS): 1. Xerostomia 2. Orofacial pain 3. Taste change
Amodio et al./2014	Oral health after breast cancer treatment in postmenopausal women	To characterize oral health in postmenopausal breast cancer survivors.	Brazil	Post-menopausal breast cancer survivors	Quantitative descriptive	1. Chronic periodontal disease
Qutob et al. /2013	Implementation of a hospital oral care protocol and recording of oral mucositis in children receiving cancer treatment	To implement a standardized hospital oral care protocol and record the incidence of oral mucositis for inpatients with childhood cancer.	Australia	Pediatric patients with cancer	Quantitative descriptive	1. Mucositis
Velten et al./2017	Prevalence of oral manifestations in children and adolescents with cancer submitted to chemotherapy	To evaluate changes in oral lesions during follow-up of children and adolescents in chemotherapy	Brazil	Children and adolescents with cancer	Quantitative descriptive	1. Mucositis 2. Xerostomia 3. Cold sores 4. Candidiasis
Ezenwa et al./2016	Caregivers’ perspectives on oral health problems of end-of-life cancer patients	To determine caregivers’ perspectives on oral health problems in cancer patients at the end of life and explore factors that contribute to those perspectives.	United States of America	Advanced cancer patients	Quantitative descriptive	1. Xerostomia 2. Orofacial pain 3. Taste change
Mercadante et al./2015	Prevalence of oral mucositis, dry mouth, and dysphagia in advanced cancer patients.	To determine the prevalence and the characteristics of oral symptoms in a large population of advanced cancer patients.	Argentina	Advanced cancer patients	Quantitative descriptive	1. Mucositis 2. Dry mouth 3. Dysphagia
Matsuo et al./2016	Associations between oral complications and days to death in palliative care patients	To investigate the associations between the incidence of oral problems and the days to death (DTD) in patients receiving palliative care.	Japan	Patients receiving palliative care	Quantitative descriptive	1. Dental caries 2. Gingival inflammation 3. Tongue coating 4. Candidiasis 5. Tongue inflammation 6. Dry mouth 7. Bleeding spots
Kvalheim et al./2016	End-of-life palliative oral care in Norwegian health institutions. An exploratory study.	To explore circumstances surrounding procedures and knowledge regarding oral care for terminal patients in Norwegian healthcare institutions.	Norway	Nurses for end-of-life patients	Quantitative descriptive	1. Dry mouth 2. Plaque 3. Food particles and fungus Infections 4. Sores and scab 5. Viscous ropy saliva and chapped lips 6. Reduced appetite and pain 7. Dysphagia 8. Halitosis 9. Coughing and problems using dentures
Bogaardt et al./2015	Swallowing problems at the end of the palliative phase: incidence and severity in 164 unsedated patients.	To establish the incidence of swallowing problems and related problems in the dying phase	Netherlands	Dying patients	Quantitative descriptive	1. Swallowing problems 2. Frequent coughing 3. Problems with oral secretions
Meidell et al./ 2009	Acupuncture as an optional treatment for hospice patients with xerostomia: an intervention study	To investigate the feasibility of conducting a 5-week acupuncture intervention in a hospice, and the effect of acupuncture on xerostomia, dysphagia and dysarthria in patients with terminal cancer.	Sweden	End-of-life patients	Quantitative comparative	1. Xerostomia 2. Dysphagia 3. Dysarthria
Lagman et al./2017	Single-Dose Fluconazole Therapy for Oral Thrush in Hospice and Palliative Medicine Patients.	To assess the efficacy of a single-dose fluconazole 150 mg for oral thrush.	United States of America	Palliative and hospice patients with advanced cancer and have a clinical diagnosis of oral thrush	Quantitative descriptive	1. Oral thrush
Momo et al., 2017	Assessment of indomethacin oral spray for the treatment of oropharyngeal mucositis-induced pain during anticancer therapy	To assess the efficacy and safety of indomethacin (IM) oral spray (OS) as a pain control therapy for oropharyngeal mucositis due to anticancer chemo- and radiotherapy	Japan	Patients with head and neck carcinomas and haematological tumours	Quantitative comparative	1. Mucositis
Ling & Larsson/ 2011	Individualized pharmacological treatment of oral mucositis pain in patients with head and neck cancer receiving radiotherapy	To assess the effect of pharmacological treatment in head and neck cancer patients with OM-induced pain and swallowing difficulties.	Sweden	Patients with head and neck cancer undergoing radiotherapy	Quantitative descriptive	1. Mucositis 2. Pain 3. Swallowing difficulties
Gligorov et al./2011	Prevalence and treatment management of oropharyngeal candidiasis in cancer patients: results of the French CANDIDOSCOPE study.	To evaluate the prevalence of oropharyngeal candidiasis (OPC) in cancer patients treated with chemotherapy and/or radiotherapy.	France	Cancer patients treated with chemotherapy and/or radiotherapy	Quantitative descriptive	1. Oropharyngeal candidiasis 2. Mucositis 3. Xerostomia 4. Taste changes 5. Pain
Davies et al./2006	Oral candidosis in patients with advanced cancer	To determine the epidemiology, aetiology, clinical features and microbiological aspects of oral candidosis in a cohort of cancer patients receiving specialist palliative care.	United Kingdom	Cancer patients receiving specialist palliative care.	Quantitative descriptive	1. Oral yeast carriage 2. Oral candidiasis 3. Xerostomia
Wilberg et al./2012	Oral health is an important issue in end-of-life cancer care.	To assess the prevalence of oral morbidity in patients receiving palliative care for cancers outside the head and neck region and to investigate if information concerning oral problems was given.	Norway	Cancers patients outside the head and neck region	Qualitative interview	1. Xerostomia 2. Mucosal friction 3. General oral discomfort 4. Taste changes 5. Candidiasis
Rydholm & Strang/2002	Physical and psychosocial impact of xerostomia in palliative cancer care: a qualitative interview study	To explore the global effects of xerostomia, with a specific focus on psychological and social consequences.	Sweden	Patients with advanced malignancies and symptomatic xerostomia	Qualitative interview	1. Subjective discomfort 2. Dryness or burning sensation 3. Loss of function e.g. articulation or swallowing 4. Increased infection (oral thrush and ulcerations)
Rohr et al./2010	Oral discomfort in palliative care: results of an exploratory study of the experiences of terminally ill patients.	To examine oral discomfort from the perspective of terminally ill patients.	Australia	Terminally-ill patients	Qualitative interview	1. Xerostomia 2. Bouts of ulceration and infection

### Common oral conditions among palliative patients

Out of 19 studies, 13 reported xerostomia or dryness of mouth [5, 11, 14–17, 19, 23–28]. Eight studies reported candidiasis or oral thrush [11, 14, 16, 20, 23–26]. Six studies reported dysphagia or swallowing difficulties [15, 17–19, 22, 26]. Five reported mucositis [13–15, 21, 22] and 5 orofacial pain [5, 17, 22, 23, 28]. Four studies reported taste changes [5, 23, 25, 28] and two reported ulceration [5, 26], coughing [17, 18] and oral discomfort [25, 26].

Other oral conditions reported are stomatitis [11], salivary hypofunction [5], mucosal erythema [5], fungal infection [5], periodontitis [12], cold sores [14], dental caries [16], gingival inflammation [16], tongue coating and inflammation [16], bleeding spots [16], plaque [17], food particles and fungus infection [17], sores and scabs [17], viscous ropy saliva and chapped lips [17], halitosis [17], problems using dentures [17], problems with oral secretions [18], dysarthria [19], oral yeast carriage [24], mucosal friction [25], and bouts of ulceration and infection [27].

### Multifaceted impact of oral conditions in palliative patients

Table 3 presents our findings associating the social and functional impact of oral conditions [5, 25–28]. Our review also revealed social and functional impact of having oral conditions among palliative patients. Social impact include feeling worried, bothered, a feeling of less satisfying life, shame, anxiety, depression, increased feelings of being a patient rather than a person, not wanting people to be around them which affected their social interaction and resulted in loneliness [5, 25–28]. Functional impact include difficulties in swallowing, speaking and eating, food restriction, sense of oral dryness and pain, which resulted in lack of food enjoyment [5, 26, 28].

**Table 3:** Studies addressing the impact of oral conditions among palliative patients

Study	Findings associating oral conditions and its social and/or functional impact	
	Social	Functional
Fischer et al.	Orofacial pain and salivary hypofunction had significant associations with social impact (p < 0.001) such as worrisome that affected the patients’ social interactions.	Xerostomia (*p* < 0.001), orofacial pain (*p* < 0.001), salivary hypofunction (*p* < .001) and taste change (*p* = 0.042) had significant associations with functional impact, which was possibly related to food enjoyment.
Ezenwa et al.	Xerostomia, orofacial pain and taste change had social impact of feeling worried, bothered, not wanting people around and a feeling of less satisfying life, with percentage agreement ranged from 41 to 64% between caregivers and care recipients. However, a significant difference in the means of the social impact subscale was reported between the two groups (*p* = 0.02), with caregivers overestimating social impact.	Xerostomia, orofacial pain and taste change had functional impact which include swallowing difficulty, speaking difficulty, eating difficulty, food restriction, dryness and pain, with significant correlation between caregivers’ and care recipients’ ratings (*p* < 0.001)
Rydholm & Strang	Xerostomia was reported to have psychosocial effects, including shame, increased feelings of being a patient rather than a person and a tendency to avoid social contact, resulting in loneliness.	Xerostomia was reported to be associated with loss of oral function, such as in articulation and swallowing.
Rohr et al.	Orofacial pain prevent patient from sharing and enjoying meals with friends and family, which limit their social outings and participation at special occasions. Participants were more ‘tentative’ in holding a conversation with others due to speech difficulties, hence avoiding ‘close physical contact’ with their loved ones.	Xerostomia was described as ‘constantly there’, causing swallowing difficulties and loss of taste. Difficulty of swallowing was also described as ‘unbearable at times’.
Wilberg et al.	Xerostomia and taste alterations were associated with anxiety (*p* = 0.04) and depression (*p* = 0.34)	n/a

*n/a not available

### Management of oral conditions among palliative patients

Table 4 presents our findings on the management of some oral conditions among palliative patients and their effectiveness [11, 15, 17, 19–23] Our study revealed that the common management options for xerostomia are drug and medical treatments [15], lubricating lips and mucosa [17], acupuncture [19], and standard oral care which improved dry mouth (in 80% or more) of the patients [11]. For candidiasis, a single-dose fluconazole 150 mg via mouth was found to be very effective as the symptoms decreased significantly (*P* < 0.001) in most patients [20], and local antifungal treatments were reported to be efficacious in 78.1% of the patients [23]. A substantial improvement of dysphagia was also observed after fifth treatment of acupuncture [19], however, its management using step-based pharmacological intervention and topically acting drugs caused worsening of swallowing and soreness of mouth [22]. Also, the management of mucositis using step-based pharmacological intervention and topically-acting drugs did not improve the oral condition [22], however, an indomethacin oral spray has been proven to relieve pain after 25 min [21].

**Table 4:** Studies addressing the management of oral conditions among palliative patients

Oral condition	Study	Management	Effectiveness
Xerostomia	Mercadante et al.	Drug medication • Opioids • Corticosteroids • Diuretics • Benzodiazepines • Anticonvulsants • Neuropletics • Nonsteroidal anti-inflammatory drugs Medical treatment • Chlorexidine • antifungal drugs • Benzydamine • Natural agents	n/a
	Kvalheim et al.	Lubricating lips • Eucerin liniment • Glycerol • Vaseline • Blisex • Lypsyl • Lip stick • Lip cream Lubricating mucosa • Glycerol • Glycerol solution 17% • Glycerol solution 50% • Glycerol solution 70% • Glycerol with peppermint oil • Glycerol and Chlorhexidine • Xylocaine/Lidocaine viscous • Xylocaine/Lidocaine viscous • Paracetamol mixture and cream • Panodil mixture and cream 1:1 • Pure cream • Zendium saliva • Zendium gel • Groundnut oil • Saliva gel • Oralbalance • Mouth moisturiser	n/a
	Meidell & Rasmussen	Acupuncture treatment twice a week for 5 weeks – a total of ten treatments.	Measurements were using visual analogue scale (VAS), consisted of a horizontal line, 0–10 cm, where 0 represented no problem or discomfort and 10 represented severe problems and discomfort. The feeling of dryness of the mouth declined for all the participants as the series of treatment proceeded. In most cases a substantial improvement could not be noted until after fifth treatments. VAS decreased from 7.5 to 4.8 after fifth treatments (P < 0.001). Between the sixth and tenth treatments, the VAS decreased from 4.8 to 3.3 (*P* < 0.001). The VAS decreased from 7.5 before the baseline to 3.3 before the tenth treatment (P < 0.001).
	Nakajima	Standard oral care by nursing staff of the wards, which include moisturizing, brushing, and oral cleaning (such as tongue coating removal) or oral massage performed by ward staff on a regular basis to resolve dry mouth). Intervention by specialist oral care team (specialist oral care) was performed as needed.	The rate of dry mouth improvement by oral care intervention was investigated by the severity (Grade 1, 2 and 3). All grade 1 cases were improved by standard oral care (100%). Grade 2 dry mouth was improved by standard oral care in 85% in good oral intake group (oral food intake was 30% or more) and 71% in poor oral intake group (oral food intake was less than 30%). Six ineffective cases of poor oral intake group were treated with specialist oral care, resulting in an improvement rate of 83%. Grade 3 dry mouth was improved by standard oral care in 40% in good oral intake group, and 2 ineffective cases were treated with specialist oral care, resulting in an improvement rate of 80%. In poor oral intake group, improvement was achieved by standard oral care in 67%, and 8 ineffective cases were treated with specialist oral care, resulting in an improvement rate of 81%. Thus, these interventions improved dry mouth in 80% or more of the patients both in good oral intake group and in poor oral intake group.
Candidiasis	Lagman et al.	A single-dose fluconazole 150 mg via mouth	Majority had complete response, except 2 who did not respond to treatment. Probable side effects of the medication included nausea in 4 patients, abdominal pain in 1, and diarrhea in 1. Both the change in the number of symptoms and the symptom scores before and after treatment decreased significantly (P < 0.001).
	Gligorov et al.	Local antifungal treatments were prescribed in 123 (75%) patients. Amphotericin B mouthwashes were administered in 67 (54.5%) patients, miconazole mucoadhesive buccal tablet in 36 patients (29.3%), and nystatin mouthwashes in 20 (16.3%) patients. Fluconazole, an oral systemic treatment, was prescribed in 41 (25%) patients at a dosage of 50 mg/day, 100 mg/day, and 200 mg/day in 7 (17.7%), 22 (53.7%), and 10 (24.4%) patients, respectively. Concomitant non-antifungal treatments were prescribed in 57 (35%) patients, mainly sodium bicarbonate mouthwashes in 45 patients.	Miconazole MBT was reported to be “efficacious” or “very efficacious” in 25 of 32 patients (78.1%) vs. 39 of 51 (76.5%) for amphotericin B, and 9 of 15 60%) for nystatin. The nonefficacy reported by the patients was related to noncompliance to treatment; 30% of noncompliant patients vs. 14.3% of those compliant rated amphotericin B as “slightly efficacious or not efficacious.”
Dysphagia	Meidell & Rasmussen	Acupuncture treatment twice a week for 5 weeks – a total of ten treatments.	Measurements were using visual analogue scale (VAS), consisted of a horizontal line, 0–10 cm, where 0 represented no problem or discomfort and 10 represented severe problems and discomfort. A substantial improvement of dysphagia was not obvious until after fifth treatments when the VAS had decreased from 5.6 to 4.1 (*P* < 0.001). Between the sixth and tenth treatments, the VAS decreased from 4.1 to 3.7 (*P* = 0.81). The VAS decreased from 5.6 before the baseline to 3.7 before the tenth treatment (*P* = 0.01).
	Ling & Larsson	Step-based pharmacological intervention 1. Acetaminophen 2. NSAID 3. Opioids 4. Adjuvant medication - Amitryptilin, gabapentin, or pregabalin were considered due to neurotic pain, mainly tumor-related. - Betametasone was considered for optimized anti-inflammatory effect, impaired general condition, or antiemetic effect. Topically acting drugs - Lidocain and benzydamine were prescribed at RT start by the dental services at the hospital to all patients with an irradiated mouth.	Soreness in the mouth showed unexpectedly significant worsening (*P* = 0.001) between baseline (TQ1) and 1 week later (TQ2). Significant worsening was found for three swallowing questions about liquids (*P* = 0.007) and solid food (*P* = 0.004), choking when swallowing (*P* = 0.018).
Mucositis	Ling & Larsson	Step-based pharmacological intervention 1.Acetaminophen 2.NSAID 3.Opioids 4.Adjuvant medication - Amitryptilin, gabapentin, or pregabalin were considered due to neurotic pain, mainly tumor-related. - Betametasone was considered for optimized anti-inflammatory effect, impaired general condition, or antiemetic effect. Topically acting drugs Lidocain and benzydamine were prescribed at RT start by the dental services at the hospital to all patients with an irradiated mouth.	Four oral mucositis (OM) grades were used: 0: No reaction 1: Hyperaemia, impressions, soreness, edema 2: Erythema, occasional ulcers, soreness 3: Painful erythema, larger fibrin-coated ulcers 4: Widespread ulcerated areas, easily bleeding, very painful In the early intervention (EI) group, the OM grade increased between baseline (TQ1) and 1 week later (TQ2) (P < 0.001). In the late intervention (LI) group, the OM grade was unchanged between TQ1 and TQ2 (*P* = 0.059).
	Momo et al.	Indomethacin (IM) oral spray (OS)	Pain relief was achieved in 93% patients at 25 (5–60) min after applying the IM-OS preparation (15.6 ± 3.4 μg/kg) and analgesic effects were maintained for 120 (10–360) min. The pain was significantly decreased after using the spray (3.6 ± 0.7 vs. 2.4 ± 0.9, *p* < 0.01). Moreover, urinary IM excretion rates after applying the IM spray preparation were 1.8 ± 0.8% of the IM oral spray dose (130.5 ± 77.7 μg/kg/day), which was markedly lower than that following oral administration of IM (60%). No adverse events were observed following application of the spray.

*n/a not available

### Treatment challenges of oral conditions in palliative patients

Table 5 presents our findings on the challenges in treating oral conditions among palliative patients [17, 18]. Only 2 of the included papers addressed the challenges in treating oral conditions among palliative patients. Kvalheim et al. (2016) found that some of the challenges were the lack of knowledge/routine, patient cooperation, resources, priority given to oral problems, as well as difficulty in accessing the mouth and retching. Bogaardt et al. (2015) observed underestimation of reported oral problems among palliative patients by rating significantly lower incidence and severity problems by the nursing staff compared to the patients’ relatives.

**Table 5:** Studies addressing the challenges in treating oral conditions among palliative patient

Study	Challenges
Kvalheim et al.	• Lack of knowledge/experience/routine (43%) • Lack of patient cooperation (38%) • Oral problems were not prioritised (22%) • Difficult access to the mouth (11%), • Lack of resources by (8%) • Retching (3%).
Bogaardt et al	• Nursing staff rated the incidence and severity of swallowing problems lower than the relatives (*p* < 0.0001) • Nursing staff rated the median severity of frequent coughing (*p* = 0.012) and loss of appetite (*p* = 0.001) significantly lower compared to the relatives’

## Discussion

To our knowledge, this review is first of its kind to systematically and comprehensively synthesize the published evidence on oral conditions among palliative patients, impact, management and challenges in the management. Our review found that the most common oral conditions among palliative patients are xerostomia, oral candidiasis, dysphagia, mucositis, orofacial pain, taste change and ulceration. A previous study by Saini et al. (2009) has also stated xerostomia, oral candidiasis, mucositis, dysphagia, ulceration, taste disorders and pain as the most common oral problems among palliative patients [2]. Another discussion paper on oral cavity complications of patients with advanced cancer also found that xerostomia, oral candidiasis and taste alterations are very common among these patients which could lead to malnutrition and communication disorder [29]. In addition, Mulk et al. (2014) described the role of dentist in palliative team and categorized xerostomia and trouble in swallowing as the indication of terminal phase of life [30]. Chen (2015) proposed an oral health care model for seriously-ill old people and stated that xerostomia is a major problem in all dying stages (decline, pre-active dying and actively dying) which worsen with each stage due to kidney failure, dehydration, and the use of anticholinergic medications during the actively dying phase [31].

Our review also revealed social and functional impact of having certain oral conditions among palliative patients. In agreement, Saini et al. (2009) stated that oral lesions have an immense impact on the quality of life of patients with complex advanced diseases, causing considerable morbidity to patient’s physical condition due to reduced oral intake and weight loss, as well as psychological well-being due to impaired communication and feelings of exclusion and social isolation. Mulk et al. (2014) explained that the most common psychological problem for the elderly requiring a palliative approach is depression, and due to the lack of proper oral hygiene among depressed patients, they often present with halitosis (bad breath) which may cause people around them to stay away from them, causing severe social impact among these patients.

Our review also reported various treatment options for several oral conditions. For example, using salivary substitutes for xerostomia, and using fluconazole for candidiasis, and its effectiveness among palliative patients. Saini et al. (2009) also reported similar treatment options for some oral conditions as in the present study, they highlighted that one of the management option for dysphagia is to remove coating or plaque from teeth, and removal of dental prosthesis to clean and rectify for any technical error for mucositis, whilst emphasizing that the management of oral problems in palliative patients should be carried out as a team work and treatment protocol should be available to guide non-dentist and dental expert. On the other hand, a clinical paper on the management of oral mucositis in cancer patients found that the current clinical management of mucositis is largely focused on palliative measures such as pain management, nutritional support and maintenance of good oral hygiene, with several promising therapeutic agents in various stages of clinical development [32]. However, none of the studies mentioned complimentary therapies such as acupuncture as a treatment option neither for xerostomia and dysphagia nor any oral conditions among palliative patients.

Our review also highlights that the lack of knowledge among healthcare providers posed a challenge in treating oral conditions among palliative patients. A study reported that training and involvement of dental professionals in caring for palliative patients seem to remain limited [33]. On the other hand, evidence also report that patients and their families are less likely to prioritize oral care needs due to increased diseases burden, transportation difficulties and psychological distress at the end of life [4]. This study also found patient cooperation as a challenge in treating oral conditions among palliative patients as it was explained that is due to the process of transferring palliative patients to dental offices for oral examination and treatment that could be physically challenging and stressful for these patients.

Apart from the above, it can be seen that among the scientific articles included in the literature review, two papers are concerning head and neck cancer patients [21], which may be more significant on the patient’s oral well-being conditions, both for the localization of the tumor and the regional radiotherapy. Therefore, future reviews can focus on patients with specific types of cancer and their oral conditions. This would greatly contribute to the body of knowledge on palliative care. Regardless, our review has provided baseline knowledge that can guide health care professionals in palliative settings.

## Conclusion

This review summarizes the diverse oral conditions that challenge the quality of life of palliative patients. Evidence is emerging on various treatment options for management of oral conditions among diverse palliative conditions. Our review also highlights the lack of evidence investigating palliative oral care among specific group of patients such as patients with dementia, geriatric or pediatric advanced cancer patients. Yet, this review provides baseline comprehensive knowledge and practice of quality oral care for palliative patients that may guide health care professionals in palliative settings.

## Data Availability

The datasets used for the current study are available from the corresponding author on reasonable request.

## Abbreviations

PRISMA: Preferred Reporting Items for Systematic Reviews and Meta-Analyses
JBI: Joanna Briggs Institute
